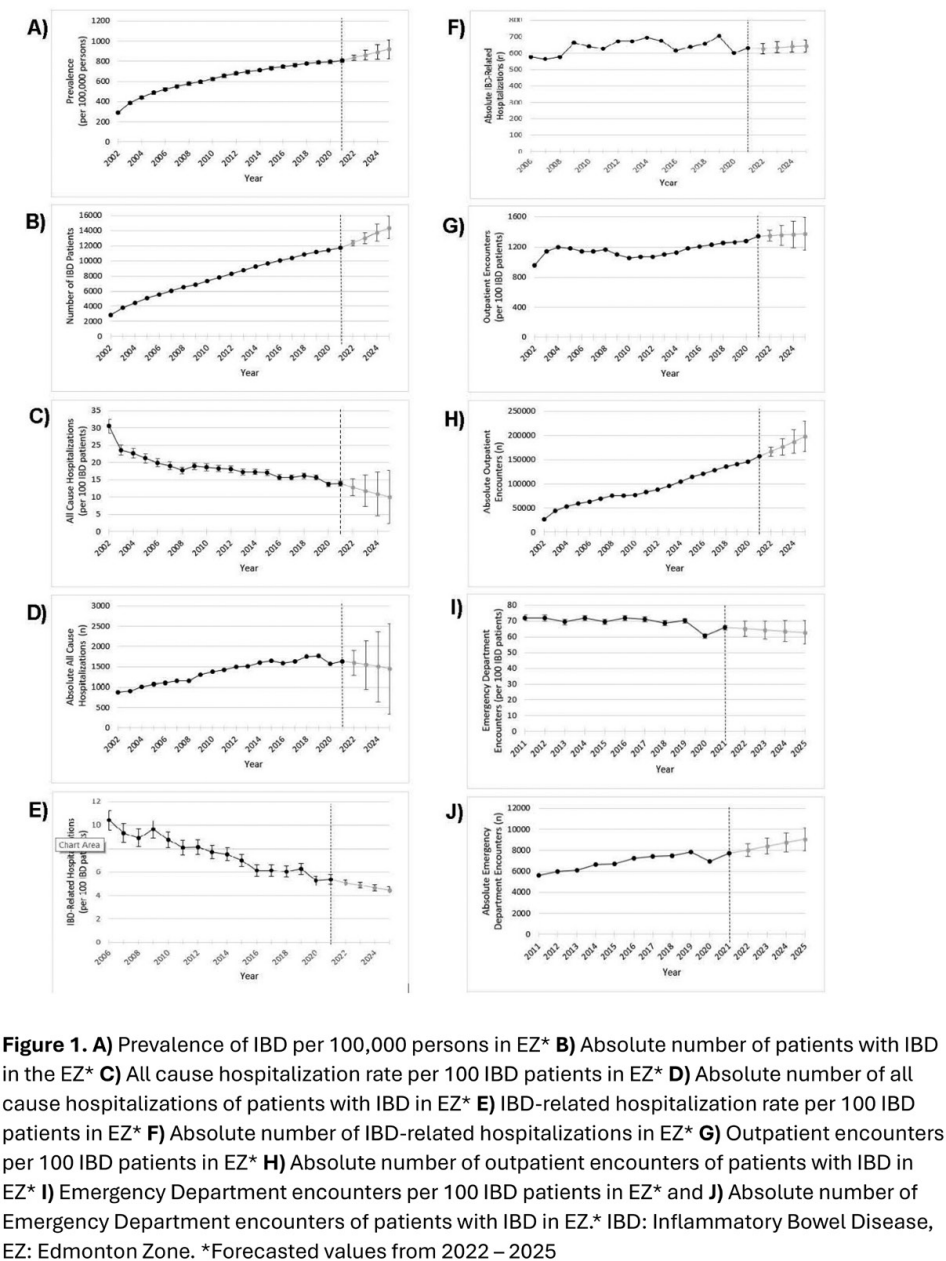# Poster Session I - A106 PREVALENCE AND HEALTH CARE ENGAGEMENT FOR INFLAMMATORY BOWEL DISEASE PATIENTS IN THE EDMONTON ZONE: PAST, PRESENT, AND FUTURE

**DOI:** 10.1093/jcag/gwaf042.106

**Published:** 2026-02-13

**Authors:** J Cooper, S Coward, J Bakal, C Wong, G G Kaplan, F Hoentjen

**Affiliations:** Adult Gastroenterology, University of Alberta Faculty of Medicine & Dentistry, Edmonton, AB, Canada; University of Calgary Cumming School of Medicine, Calgary, AB, Canada; University of Calgary Cumming School of Medicine, Calgary, AB, Canada; Adult Gastroenterology, University of Alberta Faculty of Medicine & Dentistry, Edmonton, AB, Canada; University of Calgary Cumming School of Medicine, Calgary, AB, Canada; Adult Gastroenterology, University of Alberta Faculty of Medicine & Dentistry, Edmonton, AB, Canada

## Abstract

**Background:**

Inflammatory bowel disease (IBD) is a chronic inflammatory condition with increasing prevalence in Canada.

**Aims:**

We assessed healthcare utilization over time among individuals with IBD to identify trends and inform the need for a national level needs assessment.

**Methods:**

Individuals with IBD in the Edmonton Zone were identified from the population-based Alberta IBD Surveillance Cohort using administrative data from 2002-2021 The absolute number and prevalence of IBD, along with the number and rates of emergency department encounters, hospitalizations, and outpatient encounters are reported. Annual average percent change was calculated, and Auto-Regressive Integrated Moving Average models forecasted each parameter with 95% prediction intervals (PI).

**Results:**

From 2002 to 2021, the number of individuals with IBD increased from 2,855 to 11,709, with a forecasted 5-fold increase to 14,416 (95% PI: 12965 – 15867) cases by 2025. IBD prevalence rose from 292 to 806 per 100,000 persons by 2021, with a forecasted 3.2-fold increase to 922 per 100,000 persons (95% PI: 828.7 – 1014.2) by 2025. All cause hospitalization rates declined from 30.5 to 13.9 per 100 IBD patients, but the absolute number increased from 872 to 1,636. Outpatient encounters rose from 27,384 to 157,181 by 2021 with a forecasted 7.24-fold increase to 198,320 (95% PI: 16,7014 – 22,9626) by 2025. Emergency department encounters increased from 5,614 to 7,703 by 2021.

**Conclusions:**

The burden of IBD on the health care system is rising in the Edmonton Zone due to a rapidly increasing prevalence, highlighting the need for national evaluation to inform resource allocation and maintain high-quality IBD care.

**Funding Agencies:**

None